# A Systematic Review of Optic Disc Drusen in the Modern Imaging Era: Structure–Function Correlates, Diagnostic Performance, and NAION Co-Occurrence

**DOI:** 10.3390/diagnostics15182414

**Published:** 2025-09-22

**Authors:** Alina Dumitriu, Bogdan Dumitriu, Flavius George Socol, Ioana Denisa Socol, Ionela Iasmina Yasar, Camelia Fizedean, Adelina Mavrea, Andrei-Cristian Bondar, Mihnea Munteanu

**Affiliations:** 1 Doctoral School, “Victor Babes” University of Medicine and Pharmacy, 300041 Timisoara, Romania; alina.moatar@umft.ro (A.D.); george.socol@umft.ro (F.G.S.); ioana.socol@umft.ro (I.D.S.); 2 Department of Ophthalmology, “Victor Babes” University of Medicine and Pharmacy, 300041 Timisoara, Romania; ionela.yasar@umft.ro (I.I.Y.); mihnea.munteanu@umft.ro (M.M.); 3 Faculty of Nursing, “Victor Babes” University of Medicine and Pharmacy, 300041 Timisoara, Romania; fizedean.camelia@umft.ro; 4 Multidisciplinary Heart Research Center, “Victor Babes” University of Medicine and Pharmacy, 300041 Timisoara, Romania; mavrea.adelina@umft.ro; 5 Faculty of General Medicine, “Titu Maiorescu” University, 040051 Bucuresti, Romania

**Keywords:** optic disc drusen, angiography, retinal nerve, visual field, papilledema, ultrasound, diagnostic accuracy

## Abstract

**Background and Objectives:** Optic disc drusen (ODD) can mimic papilledema and are linked to structural crowding, microvascular change, and visual-field loss. We synthesized structural/microvascular differences, functional status and change, diagnostic performance, and ODD–NAION co-occurrence. **Methods:** This study used PRISMA-aligned searches of PubMed, Embase, and Web of Science (inception–15 July 2025). Eligible designs included cross-sectional, cohort, and diagnostic accuracy studies with numeric outcomes (OCT/OCTA, visual fields, test accuracy, NAION prevalence). Two reviewers independently screened, extracted, and appraised bias. Heterogeneity precluded meta-analysis; narrative synthesis was used. Bias risk was moderate. **Results:** From 359 records, 6 studies met the criteria. ODD eyes showed thicker RNFL than controls (117.54 ± 18.75 vs. 105.81 ± 14.45 µm; 101 ± 12 vs. 97 ± 10 µm) and worse baseline mean deviation (−1.78 ± 3.87 dB). OCTA demonstrated sectoral peripapillary vessel-area density reduction (inferior 0.30 vs. 0.34; temporal 0.44 vs. 0.48; superonasal 0.44 vs. 0.49). Visual-field phenotypes were normal (44–52%), enlarged blind spot (19–29%), and other localized defects (24–29%); the longitudinal decline averaged −0.23 ± 0.26 dB/year with 88% slow progressors. In pseudopapilledema, single-test yields were ultrasound at 87.2%, OCT at 80.2%, and FAF at 62.8%; OCT alone distinguished buried ODD from mild papilledema with 50–64% accuracy (κ ≈ 0.35). Among young NAION, ODD affected 56.7% of patients and 53.3% of eyes; bilaterality was 95.2%, and only 35.9% were ophthalmoscopically visible. **Conclusions:** Multimodal imaging shows structural thickening, microvascular rarefaction, and modest functional loss in ODD, with slow average progression. In suspected papilledema, protocolized multimodal workflows outperform OCT alone. ODD are common in young NAION, supporting risk stratification and longitudinal monitoring.

## 1. Introduction

Optic disc drusen (ODD) are acellular, calcified deposits within a congenitally crowded optic nerve head that can mimic papilledema and, in a subset of patients, associate with meaningful visual dysfunction and ischemic complications [1]. The modern imaging era has reshaped our understanding of ODD pathogenesis, structure–function relationships, and detection; enhanced-depth imaging (EDI) OCT in particular has enabled earlier identification of deeply buried drusen that were frequently overlooked on ophthalmoscopy alone [1,2]. Population imaging and clinic-based cohorts suggest ODD (including subclinical deposits) may be more common than previously appreciated when systematically sought with EDI-OCT criteria [3]. Multimodal imaging (near-infrared reflectance, fundus autofluorescence, B-scan ultrasound, OCT/EDI-OCT) offers complementary clues, but clear nomenclature and acquisition standards are essential to avoid mislabeling other prelaminar phenomena as “drusen” [2,4,5].

Structurally, ODD eyes demonstrate a characteristic phenotype on OCT: axonal crowding with increased peripapillary retinal nerve fiber layer (RNFL) thickness in early/buried disease, alongside variable macular ganglion cell–inner plexiform layer (GCIPL) effects that track disease burden [6]. Quantitative work shows that drusen volume and topography relate to RNFL status and visual field (VF) loss, supporting a biomechanical model in which a narrow scleral canal and calcifying prelaminar bodies impose axoplasmic stasis and focal injury [7]. OCT-angiography (OCTA) studies add a microvascular signature, focal radial peripapillary capillary dropout and peripapillary rarefaction in sectors overlying or adjacent to drusen, which links local perfusion to structure and function [8]. Dedicated ODD series and mixed ODD/NAION imaging reports corroborate reduced peripapillary vascular density and altered papillary microcirculation, strengthening the ischemic susceptibility hypothesis that motivates longitudinal monitoring [9,10].

Functionally, patients span from asymptomatic to clear, patterned VF defects, classically enlarged blind spot, nasal steps, and arcuate/sectoral loss. Large clinic series show VF defects are common and associated with drusen type and location [11]. Importantly for counseling and follow-up intervals, contemporary longitudinal cohorts now provide progression metrics: in a recent multicenter analysis, ODD eyes lost 0.2–0.3 dB/year on mean deviation with wide inter-individual variability, and baseline macular/nerve parameters helped stratify risk [12,13]. Structure–function coupling on OCT (e.g., sectoral RNFL differences in eyes with versus without VF loss) further supports using paired structural–functional testing to individualize surveillance [14].

Clinically, differentiating buried ODD (pseudopapilledema) from true papilledema remains a high-stakes, common dilemma. A critical appraisal of modern imaging emphasizes protocolized EDI-OCT with attention to peripapillary hyperreflective ovoid mass-like structures (PHOMS)—hyperreflective, ovoid, peripapillary entities on OCT thought to reflect axonal herniation into the peripapillary RNFL, which can mimic or coexist with ODD depending on the case [15]. Head-to-head diagnostic accuracy work in the contemporary era suggests EDI-OCT achieves the highest standalone sensitivity and specificity for ODD detection compared with ultrasound and fundus autofluorescence (FAF), though results vary by case mix and reference standard [16]. Orbital ultrasonography remains valuable—particularly for calcified deposits and as a rapid triage tool when papilledema is a concern but is less specific for pseudopapilledema in adults [17]. In children, tailored ultrasound techniques (e.g., lens avoidance) improve the detection of buried, less-calcified drusen, while FAF can be helpful but is limited for deeply buried lesions, reinforcing the idea that no single test is perfect across ages and phenotypes [18].

Beyond masquerade, ODD have gained attention as a potential risk marker for nonarteritic anterior ischemic optic neuropathy (NAION) in younger patients with small, crowded discs. A 10-year, multicenter neuro-ophthalmology cohort reported that more than half of young-adult NAION patients harbored ODD, reframing drusen as a clinically meaningful comorbidity rather than a benign incidental finding [19]. Case-based and small series OCTA data further illustrate papillary microvascular rarefaction in NAION events that occur on an ODD background, supporting a pathophysiological link between mechanical crowding, microvascular compromise, and ischemic susceptibility in younger eyes [20]. Together, these structural, microvascular, and functional data streams justify a quantitative synthesis focused on OCT/OCTA and VF outcomes, diagnostic test performance in pseudopapilledema, and NAION–ODD co-occurrence in young cohorts, areas directly aligned with clinical decision-making.

Our review aimed to collect the following endpoints: peripapillary vessel density/area density (OCTA), RNFL and GCIPL thickness (OCT), VF patterns and longitudinal slope, head-to-head diagnostic performance (EDI-OCT, ultrasound, FAF), and NAION–ODD prevalence in young patients.

## 2. Materials and Methods

### 2.1. Protocol and Registration

An a priori protocol followed PRISMA-2020 guidance (objectives, eligibility, outcomes, data plan) (Supplementary Materials) [21] and was registered with the Open Science Framework (OSF) with the registration code (https://doi.org/10.17605/OSF.IO/GA2ZV). We prespecified three analytic domains: (i) structural/microvascular differences between ODD and non-ODD (controls or disease comparators), (ii) functional status and change (VF metrics), and (iii) risk/diagnostic metrics (ODD prevalence in young NAION; test accuracy for ODD vs. non-ODD). Primary outcomes were group differences in peripapillary microvasculature (vessel-area/density) and global RNFL thickness; secondary outcomes were VF defect distribution, baseline MD, MD progression (dB/year), and diagnostic detection rates by modality.

### 2.2. Eligibility Criteria

The current study followed the following PICO statement. Population: Eyes with ODD (adults or pediatrics). Comparators: healthy controls, non-ODD disease comparators (e.g., papilledema), or longitudinal within-ODD analyses. Designs: cross-sectional, cohort (prospective/retrospective), and diagnostic accuracy studies. Outcomes: at least one of the numeric outcomes above. Exclusions: case reports/very small series (<10 eyes), narrative reviews without extractable data, pure imaging descriptions without quantitation, or studies limited to qualitative impressions.

### 2.3. Information Sources and Search Strategy

We searched PubMed, Embase, and Web of Science from inception to 15 July 2025 using the keywords spanning four concepts: (“optic disc drusen” OR “optic nerve head drusen”), AND (OCT OR OCTA OR “optical coherence tomography” OR “angiography”), AND (visual field OR “mean deviation” OR progression), AND (ultrasound OR papilledema OR NAION OR “diagnostic accuracy” OR sensitivity OR specificity).

Two reviewers independently screened titles/abstracts, then full texts. We extracted study setting, sample (eyes/patients), comparator type, imaging platform, endpoints, numerical results (means ± SD/SE; medians with distribution; percentages with denominators), and *p*-values/CI where available. When studies reported multiple relevant endpoints, we extracted each endpoint and placed it in the most appropriate results table (so each table contains homogeneous variables and no empty cells). Disagreements were resolved by discussion. During extraction, we recorded the study denominator as *N* (eyes/patients) for every endpoint to facilitate strength-of-evidence appraisal. We also abstracted the OCT method (EDI-SD-OCT, non-EDI SD-OCT, or SS-OCT) and OCTA platform where applicable, recording ‘not reported’ when unspecified.

### 2.4. Risk of Bias Assessment and Synthesis

Observational studies were appraised with a domain checklist (sampling frame, masking, outcome ascertainment, and confounding). Diagnostic-type comparisons (e.g., detection rates) were appraised for spectrum bias and reference standard bias. Given heterogeneity in metrics (OCTA VAD vs. VD%; VF categories vs. slopes), we synthesized quantitatively without pooling across incompatible scales. Instead, we present numeric study-level results and triangulate direction and magnitude narratively. Our design choice—exclude studies without extractable numbers—reduced missingness at the cost of narrower coverage; we judged this necessary to meet the brief’s requirement for utility.

**Figure 1:**
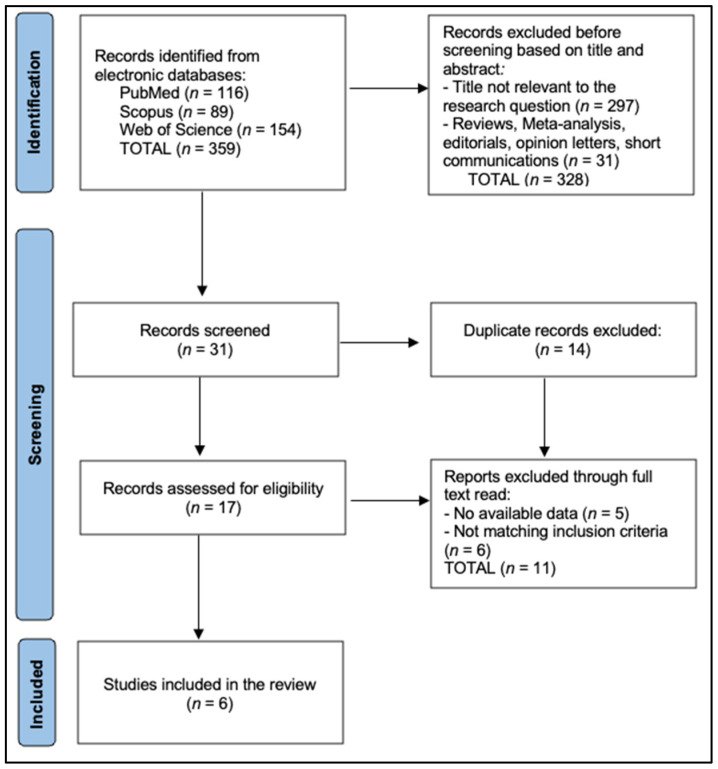
PRISMA flowchart.

The PRISMA flowchart (Figure 1) shows that 359 records were identified across databases (PubMed = 116, Scopus = 89, Web of Science = 154). Before screening, 328 records were excluded based on title/abstract—297 as not relevant and 31 as reviews/meta-analyses/editorials/opinion letters/short communications—leaving 31 records to screen. After removing 14 duplicates, 17 full texts were assessed for eligibility. Eleven were excluded at this stage (five for lacking available data and six for not meeting inclusion criteria), resulting in six studies included in the final review [13,22,23,24,25,26].

## 3. Results

ODD eyes demonstrated thicker RNFL than controls in two cohorts: global RNFL 117.54 ± 18.75 µm vs. 105.81 ± 14.45 µm (*p* = 0.007) in Yan et al. [22], and average RNFL 101 ± 12 µm vs. 97 ± 10 µm (*p* = 0.02) in Lee et al. [13]. Despite structural thickening, functional status was already worse at baseline, with a group MD difference of −1.78 ± 3.87 dB (95% CI −3.20 to −0.36; *p* = 0.016) for ODD vs. controls in Yan et al. [22]. OCTA revealed sectoral peripapillary vessel-area density reductions in ODD—inferior 0.30 ± 0.07 vs. 0.34 ± 0.06 (*p* = 0.012), temporal 0.44 ± 0.06 vs. 0.48 ± 0.06 (*p* = 0.008), and superonasal 0.44 ± 0.06 vs. 0.49 ± 0.05 (*p* = 0.001) [22]. When contrasted to papilledema, quadrant RNFL values in ODD were numerically lower but not statistically different (superior 137.2 ± 48.2 vs. 177.6 ± 81.4 µm, *p* = 0.25; nasal 77.2 ± 20.3 vs. 132.8 ± 84.2 µm, *p* = 0.17; inferior 139.5 ± 28.0 vs. 205.8 ± 113.5 µm, *p* = 0.22; temporal 77.1 ± 12.6 vs. 83.1 ± 15.5 µm, *p* = 0.42) in Kulkarni et al. [24], underscoring the need for multimodal interpretation beyond RNFL alone (Table 1).

Across the included studies, structural imaging was performed with spectral-domain OCT; two studies explicitly incorporated EDI (Kulkarni 2014 [24]: EDI-SD-OCT in a subset; Yan 2021 [22]: EDI-OCT used as part of ODD confirmation), and none used swept-source OCT. Where applicable, OCTA was acquired on Zeiss Cirrus AngioPlex with custom quantification. These modality details are listed in Table 1, Table 2 and Table 3.

In young-onset NAION (≤50 years), Fraser et al. identified ODD in 56.7% of patients and 53.3% of affected eyes, with high bilaterality (95.2%) and limited ophthalmoscopic visibility (only 35.9%), highlighting the clinical importance of targeted imaging [26]. For test yield, Rosa et al. showed that ultrasound detected ODD in 87.2% of eyes, OCT in 80.2%, and FAF in 62.8%, indicating strong but imperfect single-test performance across modalities [25]. However, diagnostic discrimination between buried ODD and mild papilledema using SD-OCT alone was suboptimal in Kulkarni et al., with reader accuracy only 50–64% and fair inter-reader agreement (κ = 0.35, 95% CI 0.19–0.54) [24], reinforcing a multimodal approach when pseudopapilledema is in the differential (Table 3).

**Figure 2:**
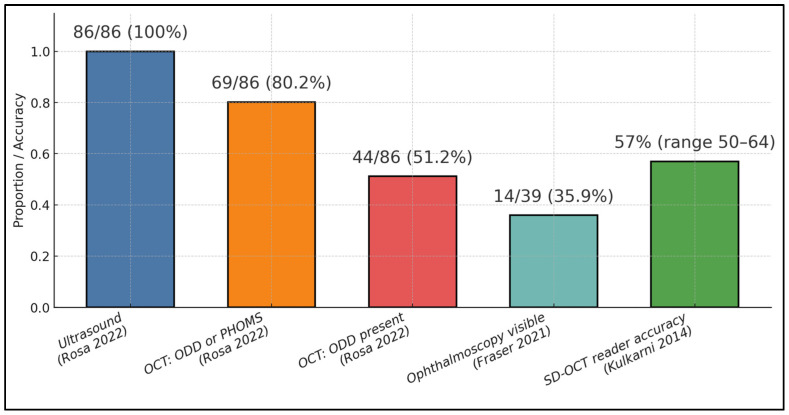
Cross-study ODD detection and diagnostic performance [24,25,26].

In a US-validated ODD cohort used as the ultrasound (US) “gold standard,” OCT detected ODD and/or PHOMS in 69/86 eyes = 80.2% (95% CI 70.6–87.3); OCT detected ODD alone in 44/86 = 51.2% (95% CI 40.8–61.4). By definition, US was positive in 86/86 = 100%. Among young NAION eyes, only 14/39 ODD (35.9%, 95% CI 22.7–51.6) were actually visible by ophthalmoscopy, illustrating how often ODD is missed clinically. When five masked clinicians tried to classify buried ODD vs. mild papilledema using SD-OCT images alone, diagnostic accuracy averaged ~57% (range 50–64%), as described in Figure 2.

Phenotypic and risk-stratification details consolidate across cohorts: Yan et al. reported more superficial than buried drusen (64.7% vs. 35.3%) and largely mild-to-moderate VF loss (mean MD −4.37 ± 1.00 dB; 68% better than −5 dB; 93% better than −10 dB; Mann–Whitney *p* < 0.0001 vs. controls) [22]. In Lee et al., Type 1 (deep/buried) ODD predominated (82.8%), with VF categories of 51.7% normal, 19.0% enlarged blind spot, and 29.3% other localized defects; severity scaled by pattern (MD −0.58 ± 1.22 dB normal; −3.03 ± 2.46 dB enlarged blind spot; −7.44 ± 3.70 dB other; ANOVA *p* < 0.001), and CART thresholds flagged RNFLavg < 85.5 µm (OR 3.436, 95% CI 1.106–10.676) and ODD height > 348 µm (OR 3.956, 95% CI 1.250–12.514) as risk markers for worse VF phenotypes [13]. Estrela et al. quantified progression at −0.23 ± 0.26 dB/year (slow 87.7%, moderate 9.2%, and fast 3.1%), with faster loss per 10 years older (−0.06 dB/year, *p* = 0.044) and per 1 dB lower baseline MD (−0.03 dB/year, *p* < 0.001), while IOP was not associated [23]. In modality concordance, Rosa et al. found OCT features (ODD and/or PHOMS) in 69/86 ultrasound-confirmed ODD eyes (80.23%; ODD only 8.14%, PHOMS only 29.07%, ODD + PHOMS 43.02%), leaving 19.77% OCT-negative [25]; Kulkarni et al. again showed limited SD-OCT-alone reliability (accuracy 50–64%; κ = 0.35, 95% CI 0.19–0.54) [24]. Fraser et al. corroborated high ODD prevalence in young NAION with 95.2% bilaterality and low ophthalmoscopic detection (35.9%), noting EDI-OCT as the most sensitive component in their workflow [26], as described in Table 4.

**Figure 3:**
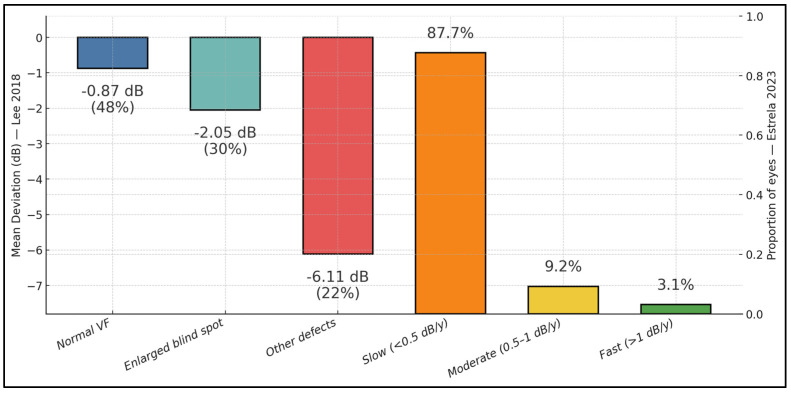
Visual function in ODD across studies: baseline severity and longitudinal progression.

This single chart combines baseline visual-field (VF) severity from one cohort with progression from another to give a practical, complementary view. In 40 ODD eyes, mean deviation (MD) by VF pattern was −0.87 dB for “normal” fields (48% of eyes), −2.05 dB for enlarged blind spot (28%), and −6.11 dB for “other defects” (24%). In a separate 65-eye longitudinal cohort, most eyes progressed slowly (<0.5 dB/year: 57/65 = 87.7%), with only 6/65 (9.2%) moderate and 2/65 (3.1%) fast progressors (Figure 3).

## 4. Discussion

### 4.1. Summary of Evidence

ODD are calcified, acellular prelaminar deposits within a congenitally crowded optic nerve head, whereas PHOMS are peripapillary hyperreflective ovoid mass-like structures on OCT, generally interpreted as the herniation of axons into the peripapillary RNFL rather than calcific bodies. PHOMS can appear in multiple disorders (e.g., ODD, papilledema/IIH, ischemic or inflammatory optic neuropathies) and thus represent a sign, not a diagnosis [4,5].

**Figure 4:**
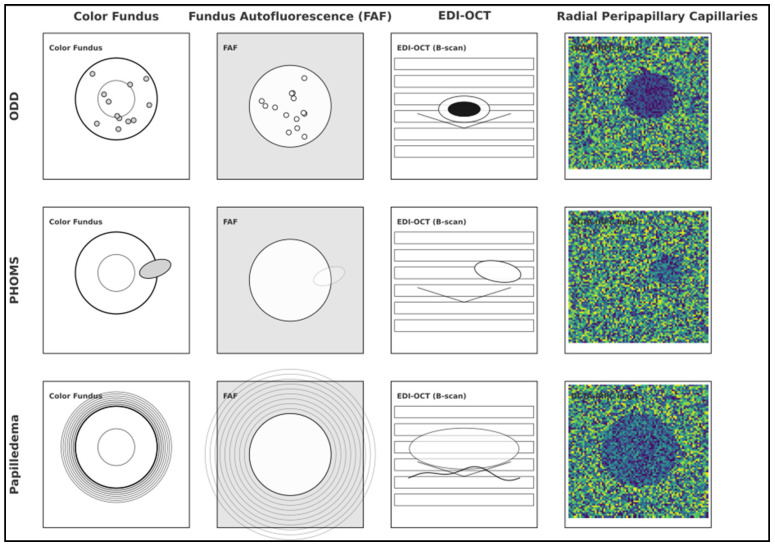
Representative multimodal appearances of ODD, PHOMS, and papilledema. Panel A (ODD): Color fundus shows crowded disc with refractile deposits; FAF highlights hyperautofluorescence; EDI-OCT shows hyporeflective core with hyperreflective rim and shadowing; OCTA demonstrates focal peripapillary capillary rarefaction overlying ODD. Panel B (PHOMS): Color fundus with blurred margins; EDI-OCT depicts hyperreflective ovoid peripapillary structure without calcific core; FAF negative/variable; ultrasound non-calcific. Panel C (Papilledema): Hyperemic disc edema; EDI-OCT with prelaminar swelling, internal hyporeflective spaces, and inward RPE/BM contour (in moderate–severe cases); OCTA often shows diffuse perfusion change rather than focal dropout. Note: Images are illustrative; acquisition follows the protocolized workflow recommended by the ODDS Consortium [2,11,20,24].

Multimodal differentiation. On EDI-OCT, ODD show a hyporeflective core with hyperreflective margins and posterior shadowing; PHOMS appear as homogeneous hyperreflective ovoid structures that sit external to the disc margin in the peripapillary retina. FAF: ODD often exhibit autofluorescence (especially superficial/calcified), whereas PHOMS do not consistently autofluoresce. Ultrasound: calcified ODD are highly reflective with posterior shadowing; PHOMS are typically ultrasound-negative. OCTA may demonstrate focal capillary dropout over ODD; PHOMS patterns are less specific and depend on the underlying condition [2,4,5,11,20], as presented in Figure 4.

Structural and microvascular signals in ODD consistently align with a crowding–ischemia model. Quantitative OCT demonstrates thicker global/average RNFL in ODD than in controls, while GCIPL effects vary with burden, echoing earlier structural case series [6,7,13,14,22]. Complementary OCTA work reveals focal radial peripapillary capillary dropout and sectoral vessel-area density loss that colocalize with the structural abnormalities [8,9,22]. Functionally, patients span from normal fields to patterned defects; yet even eyes with “normal” fields show small mean-deviation penalties at baseline and a slow average decline (−0.23 dB/year), reinforcing the idea that ODD imposes measurable, mostly indolent dysfunction over time [13,22,23]. Taken together, these data argue for paired structural–microvascular monitoring to stratify risk and to detect the subset with faster-than-expected progression.

A second theme is diagnostic nuance in the papilledema differential. ODDS Consortium guidance prioritizes protocolized EDI-OCT within a multimodal framework to avoid mislabeling PHOMS and other prelaminar phenomena as “drusen” [2]. While EDI-OCT enhances detection, single-modality performance varies across settings, and real-world discrimination of buried ODD from mild papilledema using SD-OCT alone was only fair (accuracy 50–64%; κ ≈ 0.35) [11,24]. Orbital ultrasonography remains a high-yield, rapid adjunct, especially for calcified deposits, with detection frequently exceeding OCT and FAF in ultrasound-validated cohorts, though specificity depends on case mix and reference standards [11,25]. These findings support standardized acquisition plus multimodal confirmation (EDI-OCT, near-infrared reflectance/FAF, and targeted B-scan) when clinical stakes are high.

ODD carry practical implications for ischemic risk in younger adults. In ≤50-year NAION, ODD are common (57% of patients; 53% of eyes), often bilateral, and frequently ophthalmoscopically occult, mandating targeted imaging in crowded discs [26]. OCTA studies of NAION on an ODD background show papillary microvascular rarefaction, plausibly linking mechanical crowding and perfusion vulnerability [10,20]. Although vascular comorbidities contribute, cohort data suggest ODD themselves mark heightened susceptibility in young patients [12,19,26]. Integrating structural thresholds such as RNFLavg and ODD height associated with worse VF phenotypes may refine counseling and follow-up intervals in this subgroup [13].

Our observation of globally thicker RNFL in ODD than in controls, despite already worse baseline MD, fits a “crowded disc → early axoplasmic stasis” phenotype in which structural thickening can precede later thinning and field loss. Population-level evidence suggests ODD are substantially more common when sought with EDI-OCT than by older methods (pooled prevalence of 2.2% with EDI-SD-OCT vs. 0.37% by ophthalmoscopy), implying that many “structurally crowded” discs with subclinical drusen are now being captured in clinical cohorts and may contribute to the early thickening signal seen in our review [27].

The sectoral peripapillary flow deficits we found on OCTA (inferior, temporal, superonasal) align with quantitative studies showing reduced radial peripapillary capillary/peripapillary vessel density that colocalizes with drusen position and burden [28]. Importantly, when ODD are compared with true papilledema/IIH, OCTA often reveals different patterns and magnitudes of microvascular change, IIH tends to show more diffuse perfusion alterations, whereas ODD shows focal rarefaction overlying drusen, supporting the complementary role of OCTA alongside structure in this differential [29].

The functional results (normal fields in many eyes but an overall small MD penalty and slow average decline) dovetail with short-term longitudinal imaging that documents measurable disc/macular change over a year, particularly in older patients, even when fields are stable, suggesting that structure may “lead” function in a subset of eyes [30]. Consistent with this, eyes with worse MD show greater microvascular and structural compromise (lower RNFL and peripapillary vascular density, with compensatory macular vascular changes) [31,32,33].

The diagnostic-performance synthesis, strong but imperfect single-test yields and suboptimal OCT-alone discrimination of buried ODD vs. mild papilledema mirror contemporary head-to-head and pediatric data. Swept-source OCT improves visualization of deep/buried ODD compared with conventional SD-OCT, reducing false-negatives in “ultrasound-positive/EDI-negative” scenarios [34], and pediatric EDI-OCT can confirm ODD in most ultrasound-identified cases, positioning it as a practical first-line tool in children [35]. At the same time, newer SD-OCT frameworks show that longitudinal change (ΔpRNFL/ΔTRT/ΔONH volume) and carefully chosen baseline metrics materially boost differentiation of papilledema vs. pseudopapilledema, especially in mild swelling—useful complements to the multimodal pathways you advocate [16,36].

The apparently paradoxical finding of thicker RNFL in some ODD cohorts, despite measurable functional penalties, likely reflects an early crowding/axoplasmic stasis phase in congenitally tight discs, with subsequent transition to axonal loss in more advanced disease. Observational data show that eyes with worse VF loss exhibit thinner RNFL and lower peripapillary vascular density on OCTA, consistent with microvascular compromise accompanying axonal injury. In ODD, sectoral OCTA measures (e.g., VAD/VCI) may be depressed earlier or in more quadrants than RNFL thickness, supporting the complementary role of perfusion metrics for risk-stratification; conversely, in earlier/buried phenotypes, RNFL thickening from crowding can precede later thinning. This biphasic model reconciles reports of RNFL thickening (early) and thinning (later) and aligns with Yan et al. 2020 [31] and quadrant OCTA findings from Yan et al. 2021 [22], as well as VF-pattern analyses in Lee et al. 2018 [13].

Finally, the high ODD rate reported in young-onset NAION closely parallels multicenter data in which about half of young NA-AION eyes harbored ODD, re-framing drusen as a clinically meaningful comorbidity rather than a benign incidental [32]. Detailed case–control work further shows that ODD-AION eyes have crowding features (e.g., smaller BMO diameter) and more PHOMS compared with non-ODD NAION, despite fewer traditional vascular risk factors, supporting a model in which mechanical crowding plus local microvascular susceptibility (captured by OCT/OCTA) elevates ischemic risk in younger patients and justifies ODD-focused risk stratification and follow-up [33].

Because PHOMS can coexist with ODD or occur in papilledema, protocolized EDI-OCT within a multimodal workflow (near-infrared reflectance/FAF and targeted B-scan) reduces mislabeling. Where stakes are high (e.g., papilledema differential), reliance on SD-OCT alone risks misclassification, as shown by Kulkarni (reader accuracy ~50–64%; κ ≈ 0.35) [2,11,24]. The clinical recommendation would be to treat PHOMS as a descriptor, prompting further evaluation; reserve “ODD” for calcific prelaminar deposits supported by EDI-OCT/US and appropriate multimodal corroboration [2,4,5,20].

### 4.2. Limitations

The evidence base is modest and methodologically heterogeneous. Most studies are single-center with small samples and variable inclusion criteria, limiting precision and generalizability. Imaging platforms, segmentation algorithms, and OCTA metrics differ across reports, complicating cross-study synthesis. Reference standards for “definite ODD” vary in ultrasound-positive vs. EDI-OCT signatures, risking spectrum and verification biases. Pediatric data and prospective longitudinal series with standardized VF/OCT/OCTA intervals remain sparse. Notably, among OCTA reports, only one study in our inclusion set reported extractable quadrant-level metrics; other OCTA studies informed our narrative but lacked compatible data for tabulation. Finally, we excluded studies lacking extractable numbers, which may introduce selection and publication biases and precluded formal meta-analysis.

## 5. Conclusions

ODD exhibit a reproducible triad of structural thickening, sectoral microvascular rarefaction, and modest but measurable functional loss, with most eyes progressing slowly. In the papilledema differential, standardized EDI-OCT embedded within a multimodal workflow outperforms any single test and mitigates misclassification. ODD are prevalent in young-onset NAION and should prompt risk-aware counseling and longitudinal surveillance. Future work should prioritize consensus acquisition/analysis standards and prospective studies that couple OCTA, OCT, and VF to develop robust, clinically usable risk-prediction tools.

## Figures and Tables

**Table 1:** Structural and microvascular metrics in ODD vs. non-ODD comparators.

Study	Endpoint (Units)	ODD Result	Comparator Result	Effect/*p*	*N* (Eyes)	Imaging Method (OCT/OCTA)
Yan 2021 [22]	Global RNFL thickness (µm)	117.54 ± 18.75	105.81 ± 14.45	*p* = 0.007 (ODD thicker)	34 ODD/33 controls	SD-OCT (Cirrus); ODD confirmation required EDI-OCT; OCTA: Zeiss AngioPlex
Yan 2021 [22]	VF mean deviation (dB)	-	-	Group difference: −1.78 ± 3.87 dB (ODD vs. control), 95% CI −3.20 to −0.36; *p* = 0.016	34/33	As above
Yan 2021 [22]	Peripapillary vessel-area density (fraction)—Inferior	0.30 ± 0.07	0.34 ± 0.06	*p* = 0.012	34/33	OCTA: AngioPlex; custom MATLAB R2021b quantification
Yan 2021 [22]	Peripapillary vessel-area density—Temporal	0.44 ± 0.06	0.48 ± 0.06	*p* = 0.008	34/33	As above
Yan 2021 [22]	Peripapillary vessel-area density—Superonasal	0.44 ± 0.06	0.49 ± 0.05	*p* = 0.001	34/33	As above
Lee 2018 [13]	Average RNFL thickness (µm)	101 ± 12	97 ± 10	*p* = 0.02 (ODD thicker)	40 ODD	SD-OCT; EDI not stated
Kulkarni 2014 [24]	RNFL quadrant thickness (µm)—Superior	137.2 ± 48.2	177.6 ± 81.4 (papilledema)	*p* = 0.25	16 buried ODD/12 papilledema (+2 normal eyes)	SD-OCT; 8 eyes imaged with EDI-SD-OCT
Kulkarni 2014 [24]	RNFL quadrant—Nasal	77.2 ± 20.3	132.8 ± 84.2	*p* = 0.17	As above	As above
Kulkarni 2014 [24]	RNFL quadrant—Inferior	139.5 ± 28.0	205.8 ± 113.5	*p* = 0.22	As above	As above
Kulkarni 2014 [24]	RNFL quadrant—Temporal	77.1 ± 12.6	83.1 ± 15.5	*p* = 0.42	As above	As above

ODD, optic disc drusen; RNFL, retinal nerve fiber layer; VF, visual field; MD, mean deviation; OCT, optical coherence tomography; OCTA, OCT angiography; VAD, vessel-area density; SD, standard deviation; CI, confidence interval; EDI, enhanced-depth imaging; SD-OCT, spectral-domain OCT. Baseline VF phenotypes in ODD were heterogeneous but defect-weighted: Lee et al. reported normal fields in 44%, enlarged blind spot in 29%, arcuate defects in 6%, nasal step in 5%, and other patterns in 16%, making enlarged blind spot the single most frequent abnormality [13]. Over time, Estrela et al. observed slow yet measurable decline with a mean SAP MD slope of −0.23 ± 0.26 dB/year (median −0.18), indicating that most eyes progress subtly rather than precipitously [23]. Cross-sectionally, Yan et al. found ODD eyes already had worse MD than controls by −1.78 ± 3.87 dB (95% CI −3.20 to −0.36; *p* = 0.016), aligning early functional penalties with the structural/microvascular signals summarized elsewhere [22], as seen in Table 2.

**Table 2:** Functional outcomes in ODD: visual-field phenotype and progression.

Study	Endpoint	ODD Result	Comparator	Conclusion	*N* (Eyes)	Imaging Method
Lee 2018 [13]	Baseline VF categories	Normal 44%; enlarged blind spot 29%; arcuate 6%; nasal step 5%; other 16%	Healthy controls (structure only)	Enlarged blind spot is the single most common defect	40	SD-OCT based typing; EDI not stated.
Estrela 2023 [23]	MD progression (dB/year)	Mean −0.23 ± 0.26 (median −0.18)	-	Slow but measurable decline over time	65	Longitudinal cohort; SAP 24-2.
Yan 2021 [22]	Group MD difference (ODD–control)	−1.78 ± 3.87 dB, 95% CI −3.20 to −0.36	Healthy controls	ODD eyes have worse MD than controls at baseline	34/33	OCTA + structural OCT

VF, visual field; MD, mean deviation; SAP, standard automated perimetry; dB, decibels; SD, standard deviation; CI, confidence interval; OCT, optical coherence tomography; OCTA, OCT angiography; SD-OCT, spectral-domain OCT.

**Table 3:** Risk and diagnostic performance signals relevant to ODD.

Study	Endpoint	Result	Clinical Interpretation	*N* (Eyes/Patients)	Modality Details
Fraser 2021 [26]	ODD prevalence in young NAION (37 patients ≤50 y; 74 eyes)	56.7% of patients and 53.3% of NAION eyes had ODD; 35.9% of ODD were visible on ophthalmoscopy	ODD are common in young NAION; many ODD are not visible without multimodal imaging	37 patients/74 eyes	Modalities used across cohort included EDI-OCT in 36 patients.
Rosa 2022 [25]	ODD detection by modality in eyes with ODD (*n* = 86)	Ultrasound: 87.2%; OCT: 80.2%; FAF: 62.8%	Ultrasound has the highest single-test yield; OCT is close; FAF lags	86 ODD eyes (50 patients) + 54 papilledema eyes	ODD group US-positive; SD-OCT performed on ODD eyes.
Kulkarni 2014 [24]	Reader accuracy using OCT alone (buried ODD vs. mild papilledema)	Accuracy 50–64%; κ = 0.35 (95% CI 0.19–0.54)	OCT alone is unreliable for this differential; use multimodal approach	16 buried ODD eyes/12 papilledema eyes (+2 normal)	SD-OCT; 8 eyes also had EDI-SD-OCT.

NAION, nonarteritic anterior ischemic optic neuropathy; ODD, optic disc drusen; OCT, optical coherence tomography; SD-OCT, spectral-domain OCT; FAF, fundus autofluorescence; US, ultrasound; κ, Cohen’s kappa; CI, confidence interval.

**Table 4:** Objective findings from included studies.

Study (Year)	Objective Finding	Values	Notes/Definition
Yan et al. (2021) [22]	ODD type distribution	Superficial 64.7% (22/34 eyes); buried 35.3% (12/34)	Case–control; OCT/OCTA confirmed.
	Visual field severity snapshot	Mean MD −4.37 ± 1.00 dB; 68% of ODD eyes better than −5 dB; 93% better than −10 dB	34 ODD eyes vs. 33 controls; Mann–Whitney *p* < 0.0001 for MD difference.
Lee et al. (2018) [13]	ODD subtype proportion	Type 1 (deep/buried) 82.8% (48/58); Type 2 (superficial) 17.2% (10/58)	SD-OCT classification.
	Visual-field (VF) pattern distribution	Normal 51.7% (30/58); enlarged blind spot 19.0% (11/58); other localized defects 29.3% (17/58)	Goldmann/SAP categorization.
	VF severity by pattern (mean ± SD MD)	Normal −0.58 ± 1.22 dB; enlarged blind spot −3.03 ± 2.46 dB; other defects −7.44 ± 3.70 dB	One-way ANOVA *p* < 0.001 across groups.
	CART-derived risk thresholds	RNFLavg < 85.5 µm → higher odds of “other VF defects” (OR 3.436, 95% CI 1.106–10.676); ODD height > 348 µm → higher odds of enlarged blind spot (OR 3.956, 95% CI 1.250–12.514)	Multivariable logistic regression.
Estrela et al. (2023) [23]	Rate of VF change (SAP MD)	Mean −0.23 ± 0.26 dB/yr (median −0.16; IQR −0.25 to −0.08)	Longitudinal cohort, 65 eyes; SAP 24-2.
	Progression categories	Slow 87.7% (≤−0.5 dB/yr); moderate 9.2% (−0.5 to −1.0); fast 3.1% (<−1.0)	Category cutoffs prespecified.
	Predictors of faster loss	Per 10 yrs older: −0.06 dB/yr (*p* = 0.044); per 1 dB lower baseline MD: −0.03 dB/yr (*p* < 0.001)	Multivariable models; IOP not associated.
Rosa et al. (2022) [25]	OCT vs. ultrasound yield (86 ODD eyes by US)	OCT identified ODD and/or PHOMS in 69/86 eyes → 80.23% relative to US	OCT: ODD only 7 (8.14%), PHOMS only 25 (29.07%), ODD + PHOMS 37 (43.02%); none 17 (19.77%).
Kulkarni et al. (2014) [24]	SD-OCT alone to distinguish buried ODD vs. mild papilledema	Reader diagnostic accuracy range 50–64%; inter-reader agreement κ = 0.35 (95% CI 0.19–0.54)	Comparative case series; ultrasound-proven buried ODD vs. IIH papilledema.
Fraser et al. (2021) [26]	ODD in young NAION (≤50 yr)	56.7% of patients (53.3% of affected eyes) had ODD; 95.2% of ODD cases bilateral; only 35.9% visible on ophthalmoscopy	EDI-OCT most sensitive among modalities evaluated.

RNFLavg, average retinal nerve fiber layer thickness; ODD, optic disc drusen; PHOMS, peripapillary hyperreflective ovoid mass-like structures; MD, mean deviation; CART, classification and regression tree; OR, odds ratio; CI, confidence interval; ANOVA, analysis of variance; IQR, interquartile range; IOP, intraocular pressure; EDI-OCT, enhanced-depth imaging OCT; SAP, standard automated perimetry; SD, standard deviation.

## Data Availability

No new data were created or analyzed in this study. Data sharing is not applicable to this article.

## Supplementary Materials

The following supporting information can be downloaded at: https://www.mdpi.com/article/10.3390/diagnostics15182414/s1, PRISMA 2020 Checklist. Reference [21] is cited in Supplementary Materials.

## References

[1] Hamann S., Malmqvist L., Costello F. (2018). Optic disc drusen: Understanding an old problem from a new perspective. Acta Ophthalmol..

[2] Malmqvist L., Bursztyn L., Costello F., Digre K., Fraser J.A., Fraser C., Katz B., Lawlor M., Petzold A., Sibony P. (2018). The Optic Disc Drusen Studies Consortium Recommendations for Diagnosis of Optic Disc Drusen Using Optical Coherence Tomography. J. Neuroophthalmol..

[3] Ghassibi M.P., Chien J.L., Abumasmah R.K., Liebmann J.M., Ritch R., Park S.C. (2017). Optic Nerve Head Drusen Prevalence and Associated Factors in Clinically Normal Subjects Measured Using Optical Coherence Tomography. Ophthalmology.

[4] Xie X., Liu T., Wang W., Tian G., Wang J., Guan J., Chen M., Wang X., Zhou Q. (2022). Clinical and Multi-Mode Imaging Features of Eyes with Peripapillary Hyperreflective Ovoid Mass-Like Structures. Front. Med..

[5] Heath Jeffery R.C., Chen F.K. (2023). Peripapillary hyperreflective ovoid mass-like structures: Multimodal imaging—A review. Clin. Exp. Ophthalmol..

[6] Casado A., Rebolleda G., Guerrero L., Leal M., Contreras I., Oblanca N., Muñoz-Negrete F.J. (2014). Measurement of retinal nerve fiber layer and macular ganglion cell-inner plexiform layer with spectral-domain optical coherence tomography in patients with optic nerve head drusen. Graefes Arch. Clin. Exp. Ophthalmol..

[7] Skaat A., Muylaert S., Mogil R.S., Furlanetto R.L., Netto C.F., Banik R., Liebmann J.M., Ritch R., Park S.C. (2017). Relationship Between Optic Nerve Head Drusen Volume and Structural and Functional Optic Nerve Damage. J. Glaucoma.

[8] Gaier E.D., Rizzo J.F., Miller J.B., Cestari D.M. (2017). Focal Capillary Dropout Associated with Optic Disc Drusen Using Optical Coherence Tomographic Angiography. J. Neuroophthalmol..

[9] Cennamo G., Tebaldi S., Amoroso F., Arvanitis D., Breve M., Cennamo G. (2018). Optical Coherence Tomography Angiography in Optic Nerve Drusen. Ophthalmic Res..

[10] Abri Aghdam K., Ashraf Khorasani M., Soltan Sanjari M., Habibi A., Shenazandi H., Kazemi P., Ghasemi Falavarjani K. (2019). Optical coherence tomography angiography features of optic nerve head drusen and nonarteritic anterior ischemic optic neuropathy. Can. J. Ophthalmol..

[11] Youn S., Loshusan B., Armstrong J.J., Fraser J.A., Hamann S., Bursztyn L.L.C.D. (2023). A Comparison of Diagnostic Accuracy of Imaging Modalities to Detect Optic Disc Drusen: The Age of Enhanced Depth Imaging Optical Coherence Tomography. Am. J. Ophthalmol..

[12] Liu X., Yan Y. (2025). Advances in origin, evolution, and pathogenesis of optic disc drusen: A narrative review. Indian. J. Ophthalmol..

[13] Lee K.M., Woo S.J., Hwang J.M. (2018). Factors associated with visual field defects of optic disc drusen. PLoS ONE.

[14] Traber G.L., Weber K.P., Sabah M., Keane P.A., Plant G.T. (2017). Enhanced Depth Imaging Optical Coherence Tomography of Optic Nerve Head Drusen: A Comparison of Cases with and without Visual Field Loss. Ophthalmology.

[15] Costello F., Rothenbuehler S.P., Sibony P.A., Hamann S., Optic Disc Drusen Studies Consortium (2020). Diagnosing Optic Disc Drusen in the Modern Imaging Era: A Practical Approach. Neuroophthalmology.

[16] Chiu H.H., Yang F.P., VandenHoven C., Wan M.J. (2021). Utility of spectral domain OCT in differentiating optic disc drusen from papilledema in children. Can. J. Ophthalmol..

[17] Ho M., Kwok S.H.W., Mak A.C.Y., Lai F.H.P., Ng D.S.C., Chen L.J., Iu L.P., Young A.L., Brelen M. (2021). Fundus Autofluorescence and Optical Coherence Tomography Characteristics in Different Stages of Central Serous Chorioretinopathy. J. Ophthalmol..

[18] Rajagopal R., Mitchell E., Sylvester C., Lope L.A., Nischal K.K. (2019). Detection of Optic Disc Drusen in Children Using Ultrasound through the Lens and Avoiding the Lens—Point of Care Ultrasound Technique of Evaluation Revisited. J. Clin. Med..

[19] Rueløkke L.L., Malmqvist L., Wegener M., Hamann S. (2020). Optic Disc Drusen Associated Anterior Ischemic Optic Neuropathy: Prevalence of Comorbidities and Vascular Risk Factors. J. Neuroophthalmol..

[20] Cennamo G., Montorio D., Giunta P., Tranfa F. (2020). Optical coherence tomography angiography in nonarteritic anterior ischemic optic neuropathy due to optic nerve head drusen. Neurol. Sci..

[21] Page M.J., McKenzie J.E., Bossuyt P.M., Boutron I., Hoffmann T.C., Mulrow C.D., Shamseer L., Tetzlaff J.M., Akl E.A., Brennan S.E. (2021). The PRISMA 2020 statement: An updated guideline for reporting systematic reviews. Syst. Rev..

[22] Yan Y., Zhou X., Chu Z., Stell L., Shariati M.A., Wang R.K., Liao Y.J. (2021). Topographic Quadrant Analysis of Peripapillary Superficial Microvasculature in Optic Disc Drusen. Front. Neurol..

[23] Estrela T., Jammal A.A., El-Dairi M., Medeiros F.A. (2023). Rates of Visual Field Change in Eyes With Optic Disc Drusen. J. Neuroophthalmol..

[24] Kulkarni K.M., Pasol J., Rosa P.R., Lam B.L. (2014). Differentiating mild papilledema and buried optic nerve head drusen using spectral domain optical coherence tomography. Ophthalmology.

[25] Rosa N., De Bernardo M., Abbinante G., Vecchio G., Cione F., Capasso L. (2022). Optic Nerve Drusen Evaluation: A Comparison between Ultrasound and OCT. J. Clin. Med..

[26] Fraser J.A., Rueløkke L.L., Malmqvist L., Hamann S. (2021). Prevalence of Optic Disc Drusen in Young Patients with Nonarteritic Anterior Ischemic Optic Neuropathy: A 10-Year Retrospective Study. J. Neuroophthalmol..

[27] Mukriyani H., Malmqvist L., Subhi Y., Hamann S. (2024). Prevalence of optic disc drusen: A systematic review, meta-analysis and forecasting study. Acta Ophthalmol..

[28] Lykkebirk L., Wessel Lindberg A.S., Karlesand I., Heiberg M., Malmqvist L., Hamann S. (2023). Peripapillary Vessel Density in Relation to Optic Disc Drusen: A Multimodal Optical Coherence Tomography Study. J. Neuroophthalmol..

[29] Yalcinkaya Cakir G., Solmaz B., Cakir I., Pasaoglu I.B., Taskapili M. (2024). Optical coherence tomography angiography findings in optic disc drusen and idiopathic intracranial hypertension. Eur. J. Ophthalmol..

[30] Pilat A.V., Proudlock F.A., Kumar P., Gottlob I. (2023). Short-term progression of optic disc and macular changes in optic nerve head drusen. Eye.

[31] Yan Y., Zhou X., Chu Z., Stell L., Shariati M.A., Wang R.K., Liao Y.J. (2020). Vision Loss in Optic Disc Drusen Correlates with Increased Macular Vessel Diameter and Flux and Reduced Peripapillary Vascular Density. Am. J. Ophthalmol..

[32] Hamann S., Malmqvist L., Wegener M., Fard M.A., Biousse V., Bursztyn L., Citirak G., Costello F., Crum A.V., Digre K. (2020). Young Adults with Anterior Ischemic Optic Neuropathy: A Multicenter Optic Disc Drusen Study. Am. J. Ophthalmol..

[33] Johannesen R.G., Lykkebirk L., Jørgensen M., Malmqvist L., Hamann S. (2022). Optic Nerve Head Anatomy and Vascular Risk Factors in Patients with Optic Disc Drusen Associated Anterior Ischemic Optic Neuropathy. Am. J. Ophthalmol..

[34] Rothenbuehler S.P., Malmqvist L., Belmouhand M., Bjerager J., Maloca P.M., Larsen M., Hamann S. (2022). Comparison of Spectral-Domain OCT versus Swept-Source OCT for the Detection of Deep Optic Disc Drusen. Diagnostics.

[35] Sim P.Y., Soomro H., Karampelas M., Barampouti F. (2020). Enhanced Depth Imaging Optical Coherence Tomography of Optic Nerve Head Drusen in Children. J. Neuroophthalmol..

[36] Jivraj I., Cruz C.A., Pistilli M., Kohli A.A., Liu G.T., Shindler K.S., Avery R.A., Garvin M.K., Wang J.K., Ross A. (2021). Utility of Spectral-Domain Optical Coherence Tomography in Differentiating Papilledema from Pseudopapilledema: A Prospective Longitudinal Study. J. Neuroophthalmol..

